# Eosinophilic Colitis: University of Minnesota Experience and Literature Review

**DOI:** 10.1155/2011/857508

**Published:** 2011-07-10

**Authors:** Wolfgang B. Gaertner, Jennifer E. MacDonald, Mary R. Kwaan, Christopher Shepela, Robert Madoff, Jose Jessurun, Genevieve B. Melton

**Affiliations:** ^1^Division of Colon and Rectal Surgery, Department of Surgery, University of Minnesota, 420 Delaware Street SE, Mayo Mail Code 450, Minneapolis, MN 55455, USA; ^2^Medical School, University of Minnesota, 420 Delaware Street SE, Mayo Mail Code 450, Minneapolis, MN 55455, USA; ^3^Division of Gastroenterology, Department of Medicine, University of Minnesota, 420 Delaware Street SE, Mayo Mail Code 450, Minneapolis, MN 55455, USA; ^4^Department of Laboratory Medicine and Pathology, Department of Medicine, University of Minnesota, 420 Delaware Street SE, Mayo Mail Code 450, Minneapolis, MN 55455, USA

## Abstract

Eosinophilic colitis is a rare form of primary eosinophilic gastrointestinal disease that is poorly understood. Neonates and young adults are more frequently affected. Clinical presentation is highly variable depending on the depth of inflammatory response (mucosal, transmural, or serosal). The pathophysiology of eosinophilic colitis is unclear but is suspected to be related to a hypersensitivity reaction given its correlation with other atopic disorders and clinical response to corticosteroid therapy. Diagnosis is that of exclusion and differential diagnoses are many because colonic tissue eosinophilia may occur with other colitides (parasitic, drug-induced, inflammatory bowel disease, and various connective tissue disorders). Similar to other eosinophilic gastrointestinal disorders, steroid-based therapy and diet modification achieve very good and durable responses. In this paper, we present our experience with this rare pathology. Five patients (3 pediatric and 2 adults) presented with diarrhea and hematochezia. Mean age at presentation was 26 years. Mean duration of symptoms before pathologic diagnosis was 8 months. Mean eosinophil count per patient was 31 per high-power field. The pediatric patients responded very well to dietary modifications, with no recurrences. The adult patients were treated with steroids and did not respond. Overall mean followup was 22 (range, 2–48) months.

## 1. Introduction

Primary eosinophilic gastrointestinal disease (EGID) is a rare chronic inflammatory bowel condition of unknown etiology that was originally described by Kaijser in 1937 [1]. EGID is a spectrum of gastrointestinal (GI) disorders characterized by inflammation rich in eosinophils without evidence of other known causes of eosinophilia (i.e., parasitic, infectious, drug reaction, or malignancy) [2]. The disease can affect any segment or combination of segments of the GI tract from the esophagus to the rectum, giving rise to various clinical presentations. 

Eosinophilic colitis (EC) represents the least frequent manifestation of EGID whether or not it effects other segments of the GI tract [3]. Since secondary eosinophilic inflammation may occur in numerous GI disorders such as IgE-mediated food allergy, gastroesophageal reflux disease, and inflammatory bowel disease, the true incidence and prevalence of primary EGID remains largely unknown. A recently established world-wide-web registry found that this disease mainly affects the pediatric population, although it has been reported in patients up to 68 years of age [3]. Most recently, eosinophilic esophagitis has been increasingly recognized as a distinct condition that affects about 1% of the population, both in pediatric and adult populations [4].

## 2. Patients and Methods

We searched the computerized database of the Department of Pathology at the University of Minnesota for all cases of EC occurring between 2003 and 2010. Search criteria specifically included the terms “eosinophilic colitis”, “colon eosinophilia”, and “primary eosinophilic gastrointestinal disease.” This study was approved by the Institutional Review Board of the University of Minnesota. A detailed review of each patient's medical chart was undertaken, concentrating on demographics, presentation, diagnosis, therapy, and outcome. Diagnostic criteria included a colonic biopsy showing focal aggregates of eosinophils in the lamina propria, crypt epithelium, and muscularis mucosa of at least 20 eosinophils per high-power field. Patients with evidence of secondary systemic eosinophilia or tissue eosinophilia were excluded.

## 3. Case Series

A total of five patients (3 males, 3 pediatric, and 2 adults) with a mean age of 26 years (range, 2 months to 73 years) were diagnosed with symptomatic EC (Table 1). Five patients with secondary EC were excluded. The most common symptoms at presentation were diarrhea and hematochezia. The mean duration of symptoms before pathologic diagnosis was 8 (range, 1–14) months. One pediatric patient also had selective IgA deficiency, but a pertinent workup for celiac sprue was negative. The mean eosinophil count per patient was 31 per high-power field, and eosinophil infiltration occurred in the lamina propria in all patients, with three patients also having muscularis mucosae infiltration. The most frequently observed histologic alteration was neutrophilic cryptitis in three patients (2 pediatric and 1 adult). The most common site of colonic involvement was the ascending colon. Two patients also had gastric involvement, and one patient had involvement of the rectum. The most common endoscopic findings included mucosal congestion and lymphonodular hyperplasia. Three of the five patients were treated with dietary modifications and had excellent responses with no recurrence. Of the two adult patients treated with oral steroid therapy, one had an incomplete response with only partial improvement of symptoms, and the other patient died after two months because of multiorgan failure. This last patient had full-thickness involvement of the colon as well as T-cell non-Hodgkin's lymphoma involving mediastinal and cervical lymph nodes. Pathologic evaluation on this patient revealed negative mast cell immunoreactivity for CD25 and had no evidence that malignancy was the cause of systemic or tissue eosinophilia. The overall mean followup for the entire group was 22 (range, 2–48) months.

## 4. Etiology

The etiology of primary EGID remains largely unknown. Several studies have suggested a relationship with specific food allergies. Approximately, 75% of affected patients have a history of allergy or atopy [2]. Cow's milk and soy proteins are the foods most frequently implicated in the infantile form of EC, although the condition has been described in infants exclusively breast fed or given protein hydrolyzed formulas [2]. Even less is known about the potential causes of the adult form of primary EC. A case report by Inamura et al. [5] demonstrated the accumulation of mast cells in the colon interstitium after immunohistochemical staining for mast cell tryptase, which may suggest a possible pathogenic role of IgE. Specific eosinophil chemoattractants, such as interleukin-5 and eotaxins, may also have a pathogenic role in EC [6]. Hahn and Hornick [7] specifically evaluated mast cells in mucosal biopsies from patients with systemic mastocytosis and a group of patients with diverse inflammatory disorders. They found that systemic mastocytosis patients have significantly higher densities of mast cells in aggregates or sheets on GI mucosal biopsies and positive immunoreactivity for CD25. These diagnostic criteria can be helpful in excluding patients with secondary EC.

## 5. Clinical Presentation

EC appears to have a bimodal distribution that affects neonates and young adults with no gender preference [2]. In general, EC has three hallmarks including peripheral eosinophilia, segmental eosinophilic infiltration of the colon, and functional abnormalities. 

**Figure 1:**
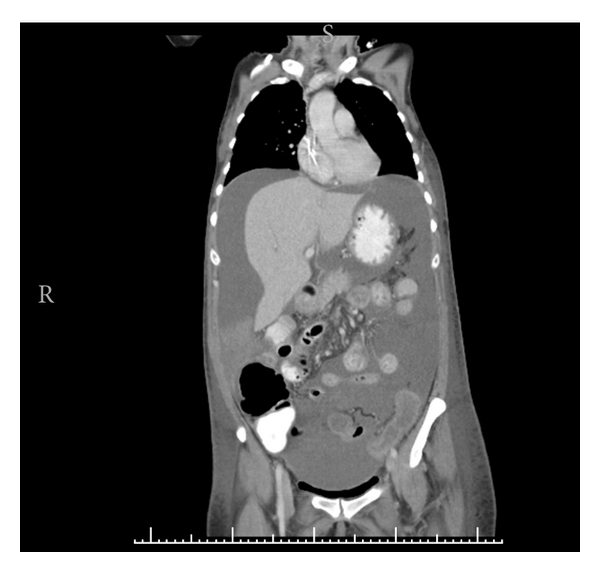
Computed tomography of the abdomen and pelvis showing colonic wall thickening and ascites in patient 4 with full-thickness eosinophilic colitis.

Symptoms and signs of EC are usually nonspecific and, depending on the affected segment, include abdominal pain, nausea, vomiting, diarrhea, bleeding, obstruction, weight loss, and ascites. In 1970, Klein et al. [8] subdivided the disease based on the layer of intestinal wall most extensively infiltrated by eosinophils. This classification provides good correlation with the physical symptoms and the pathologic findings. Mucosa-predominant disease results in diarrhea, malabsorption and protein wasting [9]. Transmural disease has been associated with bowel wall thickening on imaging studies, obstruction, volvulus [9], intussusceptions [10, 11], and even perforation [12, 13]. Serosal involvement is often distinguished by the presence of eosinophilic ascites (Figure 1) [14].

## 6. Diagnosis

The diagnosis of EC is made from the presence of gastrointestinal symptoms, peripheral eosinophilia, endoscopic and histological findings, and eosinophilic ascites, with no well-defined causes of eosinophilia on further evaluation. Twenty to 90% of patients have an increase in the eosinophil count in peripheral blood. Because some patients will not have this finding, peripheral eosinophilia is not sufficient as an initial screening tool. Radiological findings also depend on the region and layer affected and may show strictures, thickening of the bowel wall and mucosal folds [15], a rigid ileocecal valve open to reflux, and ulcerative or polypoid lesions. 

**Figure 2:**
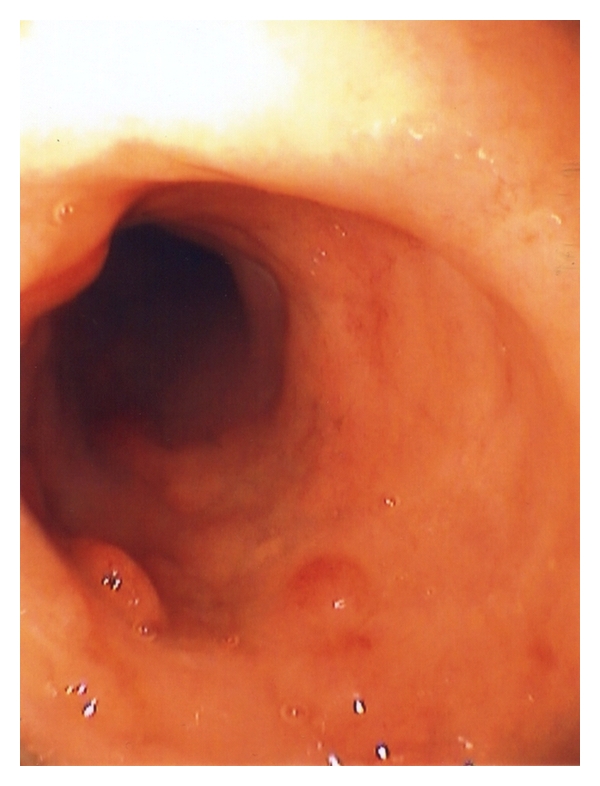
Colonoscopic view of a patient with eosinophilic colitis showing mild inflammatory changes, mucosal edema, patchy erythema, and loss of vascularity.

**Figure 3:**
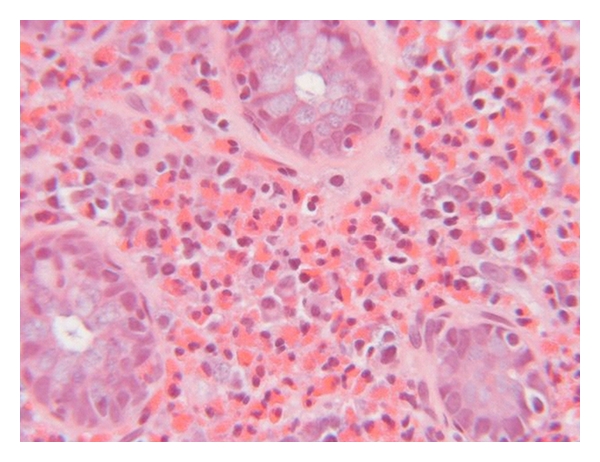
Microscopic view of the colon wall demonstrating tissue eosinophilia.

At colonoscopy, some patients have lymphonodular hyperplasia while others have endoscopic features of mild colitis including mucosal edema, patchy erythema, and loss of vascularity (Figure 2). Changes can occur throughout the colon but tend to be more prominent in the ascending colon and rectum. The only clear diagnostic criteria for any of the EGID entities pertain to eosinophilic esophagitis, which is defined by the presence of more than 15 eosinophils per high-power field in the esophageal squamous mucosa [16]. No such consensus exists for EC, although most authors have used a diagnostic threshold of 20 eosinophils per high-power field. Of note, normal values for tissue eosinophils vary widely between different segments of the colon, ranging from <10 eosinophils per high-power field in the rectum to >30 in the cecum [17], thus, location of colonic biopsy is critically important for proper interpretation of findings. Histological features include focal aggregates of eosinophils in the lamina propria, crypt epithelium and muscularis mucosa (Figure 3). 

Tissue eosinophilia in the colon may result from a number of conditions (Table 2), and EC remains a diagnosis of exclusion. Colonoscopic biopsies obtained from patients with inflammatory bowel disease, particularly with Crohn's colitis, often show severe tissue eosinophilia [18]. Parasitic infection of the colon may lead to marked eosinophilic infiltration, and repeated stool or serological testing may be needed to confirm this specific etiology. Drug-induced EC has also been described in response to several medications, including clozapine, carbamazepine, rifampicin, nonsteroidal antiinflammatory agents, tacrolimus, and gold [19–25]. Other conditions associated with EC include systemic mastocytosis, malignancy, autoimmune connective tissue disease including scleroderma, dermatomyositis and polymyositis, [9, 26, 27] allogeneic bone marrow transplantation [28], and the rare Tolosa-Hunt syndrome that features inflammatory ophthalmoparesis [29]. Idiopathic hypereosinophilic syndrome may also affect the colon, but this rare condition presents with sustained and marked peripheral eosinophilia with end-organ damage that extends beyond the gastrointestinal tract (e.g., heart and skin) [30].

## 7. Treatment

Currently, there is no level I evidence to guide treatment of EC. Therapies for EGID have been based mainly on case reports and small case series. The beneficial effect of withdrawing offending dietary triggers and prescribing elemental diets have been limited to cases with specific food allergies, especially in treating neonatal disease [31]. In contrast, the response of adults to dietary modification is less clear, and those with more severe symptoms receive some sort of pharmacologic therapy. 

Allergy testing can aid in the identification of specific antigens and the development of an elimination diet. However, allergy testing has low sensitivity and specificity with high false-positive rates and should be interpreted with caution. In a study of 35 patients with eosinophilic esophagitis who were placed on an empiric elimination diet for a 6-week trial, 74% showed improvement on esophageal biopsy [32].

Corticosteroids are the mainstay for initial management and have proven to be effective for symptom control in EC [2, 33, 34]. The majority of cases will respond within 2 weeks of treatment. Relapse is frequent and requires recurrent courses, which can lead to steroid dependence. Several studies have evaluated the efficacy of steroids in the various types of EGID and have shown significant improvement in symptom management. However, no histologic correlation has been demonstrated in EC. Also, the optimal duration of corticosteroid therapy has not yet been established. Often, a dosing strategy similar to that used in inflammatory bowel disease has been utilized. Budesonide, a synthetic oral corticosteroid, has shown to be effective in EC, particularly when the right colon and ileum are affected [35]. In a randomized, double-blind, placebo-controlled trial, Straumann et al. [36] showed that a 15-day regimen of oral budesonide was highly effective in inducing a histologic and clinical remission in adolescent and adult patients with active eosinophilic esophagitis. Oral viscous budesonide has also been found to be an effective treatment of eosinophilic esophagitis in children, improving symptoms and both endoscopic and histologic features [37]. Topical corticosteroid treatment (inhaled fluticasone) has been used in patients with eosinophilic esophagitis and has shown to be effective in symptom control but with frequent recurrences after treatment discontinuation [38, 39]. It must be emphasized that efforts to rule out parasitic or drug-induced EC are important since empiric treatment with corticosteroids may worsen the patient's condition. 

Immunomodulating therapies may provide an alternative to corticosteroids for chronic maintenance therapy. These include mast cell inhibitors, antihistamines, leukotriene receptor antagonists, and newer biologic immunotherapies. Ketotifen, an H1 antihistamine, has been shown to decrease symptoms as well as tissue eosinophilia in EGID [40, 41]. The leukotriene inhibitor montelukast, an agent that blocks the action of eosinophil chemoattractant leukotriene D4, appears also to have efficacy [42, 43]. Mast cell stabilizers, such as cromolyn, are effective by inhibiting the release of mast cell mediators such as histamine, platelet activating factor, and leukotoxin [44]. More recently, the role of immunotherapy has also been studied, with favorable outcomes reported by using monoclonal antibodies targeting interleukin 5 (mepolizumab) and IgE (omalizumab) [45, 46]. 

Currently, the only level I evidence for EGID treatment is for eosinophilic duodenitis. In a double-blinded, randomized, and controlled study evaluating the efficacy of montelukast in 40 children and adolescents, Friesen et al. [47] showed significant symptom improvement in the montelukast arm (62%) versus the placebo arm (32%) [48]. Of importance, most studies evaluating pharmacologic treatment of EC are largely observational, and medications were evaluated in combination with other treatment modalities such as dietary modifications. 

Operative treatment is only indicated for the complications of EC. These include obstruction, volvulus, intussusception and perforation. Segmental anatomic colonic resection is recommended with no clear consensus or evidence to support primary anastomosis or diversion. One must consider the degree of peritoneal contamination and overall physiologic status of the patient, as with other indications for colonic surgery. Of importance, eosinophilic ascites in the presence of colitis may be misdiagnosed as a free perforation. Depending on the patient's overall status, a diagnostic paracentesis or diagnostic peritoneal lavage may be considered initially to confirm the diagnosis and potentially avoid major surgery in an immunocompromised patient.

## 8. Conclusions

EC is a rare manifestation of EGID without a primary etiology for tissue hypereosinophilia. The pathophysiology of this condition is not well understood, but a hypersensitivity mechanism is suspected given its increased association with other atopic conditions and clinical improvement with corticosteroids. In contrast to increased trends seen in esophageal disease, the prevalence of EC does not appear to be increasing. This may suggest a different pathophysiology for EC. Symptoms are non-specific and correlate with the degree of eosinophilic infiltration of the colonic wall. The definitive diagnosis of EC is made with endoscopic biopsy demonstrating colonic tissue hypereosinophilia and the absence of any primary disorders that may cause secondary eosinophilic infiltrates. No clear consensus exists with regards to the degree of tissue eosinophilia or the presence of distinct pathologic findings. While the pediatric form of EC often subsides without intervention or after the withdrawal of atopic stimuli, the adult form may relapse and require short-term or repeated courses of steroid therapy. EC is primarily a diagnosis of exclusion but needs to be included in the differential diagnosis of many GI conditions that present with non-specific symptoms and eosinophilia. Additional studies are needed to further delineate the pathophysiology of EGID and to determine optimal treatment regimens for EC.

## Figures and Tables

**Table 1:** Case series of four patients with eosinophilic colitis.

Patient	Age gender	Symptoms	Relevant history	Colon involved	Location and mean eosinophil count per HPF	Treatment	Outcome
1	2 months-M	Hematochezia	Vesicoureteral reflux with hydronephrosis	Sigmoid	Lamina propria-23	Dietary modification	Resolution of symptoms. No recurrence
2	4 months-M	Hematochezia	GERD, C. diff colitis.	Ascending	Lamina propria-33	Dietary modification	Resolution of symptoms. No recurrence
3	3 years-M	Persistent diarrhea	Selective IgA deficiency	Ascending	Lamina propria and muscularis mucosae-38	Dietary modification	Resolution of symptoms. No recurrence
4	53 years-F	Persistent diarrhea	T-cell lymphoma (status after BMT), C. diff colitis	Ascending & rectum	Lamina propria and muscularis mucosae-29	Prednisone	Deceased after 2 months
5	73 years-F	Severe diarrhea	History of rheumatoid arthritis	Ascending and transverse colon	Lamina propria and muscularis mucosae-32	Budesonide	Incomplete response to initial treatment

HPF: high-power field, GERD: gastroesophageal reflux disease, C. diff: clostridium difficile, BMT: bone marrow transplant.

**Table 2:** Differential diagnoses of eosinophilic colitis.

Inflammatory bowel disease
Drug-induced colitis
Clozapine
Carbamazepine
Rifampicin
Gold
NSAID's
Tacrolimus
Parasitic colitis
*Enterobius vermicularis *
*Strongyloides stercoralis *
*Trichuris trichiura *
Hypereosinophilic syndrome
Systemic mastocytosis
Allogeneic bone marrow transplantation
Tolosa-Hunt syndrome

NSAID: nonsteroidal antiinflammatory drug.
